# Information quality of implant-related videos on Chinese social media platforms: a cross-sectional analysis of Bilibili, Douyin, and Xiaohongshu

**DOI:** 10.1186/s12903-026-09555-1

**Published:** 2026-08-08

**Authors:** Shuhan Gu, Ziyue Xu, Qianqian Hou, Yiming Gao, Kaixiao Yan

**Affiliations:** Center for Oral Health, Ruijin Hospital, Shanghai Jiao Tong University School of Medicine, Shanghai, China

**Keywords:** Dental implants, Social media, Health information, Patient education, Video analysis, China

## Abstract

**Background:**

As dental implant therapy becomes increasingly common, patients frequently seek implant-related information through social media platforms. However, the completeness and educational quality of such information remain uncertain, particularly on Chinese social media platforms. This study aimed to evaluate implant-related videos on three major Chinese social media platforms using complementary measures of content completeness and educational quality.

**Methods:**

A cross-sectional content analysis was conducted on implant-related videos retrieved from Bilibili, Douyin, and Xiaohongshu on June 10, 2026, using the Chinese keyword “种植牙” (“dental implant”). The first 100 videos returned by each platform’s default search function were screened, and 250 videos meeting predefined eligibility criteria were included. Video characteristics were recorded, and information quality was evaluated using the Global Quality Scale (GQS) and a newly developed Implant Content Score (ICS), a 10-domain instrument assessing the completeness of implant-related information. Differences among platforms and associations between video characteristics and information quality were statistically analyzed.

**Results:**

Of the 250 included videos, 83 originated from Bilibili, 79 from Douyin, and 88 from Xiaohongshu. Significant differences in information quality were observed among platforms. Bilibili videos showed the highest median ICS score [4 (2–5)], followed by Xiaohongshu [3 (2–4)] and Douyin [2 (1–3)] (*P* < 0.001). Similarly, Bilibili demonstrated the highest GQS score, followed by Douyin and Xiaohongshu (*P* < 0.001). Scientific accuracy was the most frequently reported content domain (97.2%), whereas comprehensive treatment planning was the least frequently reported (8.8%). Video duration was positively associated with ICS score (*r* = 0.490, *P* < 0.001), while likes, comments, and favorites showed no significant association with information quality.

**Conclusions:**

The quality of implant-related information on Chinese social media platforms varies substantially. Although Bilibili videos demonstrated relatively higher content completeness and educational quality, clinically important topics such as comprehensive treatment planning were frequently overlooked across all platforms. These findings suggest that content completeness and educational quality represent distinct dimensions of online health information quality and support the use of complementary assessment approaches. Greater involvement of dental professionals in developing evidence-based implant-related educational content may improve patient understanding and support informed treatment decision-making.

**Supplementary Information:**

The online version contains supplementary material available at https://doi.org/10.1186/s12903-026-09555-1.

## Introduction

Dental implant therapy has become a widely accepted treatment modality for the rehabilitation of partially and completely edentulous patients. Owing to its favorable long-term survival, functional performance, and esthetic outcomes [1], implant therapy is now considered a predictable and reliable option for replacing missing teeth in a broad range of clinical situations. Recent epidemiological data have demonstrated a substantial increase in implant utilization worldwide [2]. In the United States, the prevalence of dental implants among adults with missing teeth increased markedly between 1999 and 2016, reflecting the growing adoption of implant-based rehabilitation. As the demand for implant treatment continues to expand, patients are increasingly seeking information regarding implant therapy before making treatment decisions.

As the utilization of dental implants continues to increase, patients are becoming more actively involved in treatment decision-making and are increasingly seeking health-related information online. Social media platforms have emerged as important sources of medical and dental information, providing users with convenient access to educational content, professional opinions, and patient-generated experiences [3]. Previous studies have shown that a substantial proportion of patients search the internet before seeking professional consultation and often rely on online information to understand treatment options, expected outcomes, risks, and costs [4, 5]. Consequently, the quality and accuracy of health information disseminated through social media have become important concerns for healthcare professionals and researchers [3].

Several studies have evaluated the quality and reliability of oral health information available on social media platforms [6, 7]. In the field of implant dentistry, previous investigations of YouTube videos have consistently reported deficiencies in content completeness, reliability, and educational value [8, 9]. Similar findings have been reported for TikTok, where implant-related videos were found to provide low to moderate quality information and frequently lacked comprehensive clinical content [10]. However, existing evidence has been derived predominantly from English-language platforms such as YouTube and TikTok. Little is known about the quality of implant-related information disseminated through major Chinese social media platforms, despite their extensive use by Chinese patients seeking health information. Furthermore, few studies have compared multiple social media platforms using complementary measures that distinguish content completeness from overall educational quality.

Against this background, the present study aimed to evaluate the quality and content characteristics of implant-related videos available on three major Chinese social media platforms: Bilibili, Douyin, and Xiaohongshu. Specifically, the study assessed both the completeness of implant-related information using a newly developed Implant Content Score (ICS) and the overall educational quality using the Global Quality Scale (GQS). In addition, differences in information quality among platforms and the associations between video characteristics and information quality metrics were investigated. The findings of this study may provide insights into the current landscape of implant-related information on Chinese social media and help identify areas for improvement in online patient education and evidence-based communication regarding implant therapy.

## Methods

### Study design

This cross-sectional content analysis evaluated the quality and characteristics of implant-related videos available on three major Chinese social media platforms: Bilibili, Xiaohongshu, and Douyin (the Chinese counterpart of TikTok). The study aimed to assess the completeness and educational quality of implant-related information and to compare information quality across different platforms.

This study involved a retrospective analysis of publicly available videos from social media platforms. No direct interaction with human participants was undertaken, and no identifiable personal information was collected or analyzed. Because the study used only publicly available, non-identifiable data and did not involve human participants, ethics approval and informed consent to participate were not required. All procedures were conducted in accordance with relevant institutional guidelines and regulations.

### Video search conditions and strategy

Video searches were conducted on June 10, 2026, on three major Chinese social media platforms: Bilibili, Xiaohongshu, and Douyin (the Chinese counterpart of TikTok). The Chinese keyword “种植牙” (“dental implant”) was used as the search term on all platforms.

To minimize the potential influence of personalized recommendation algorithms, searches were performed using newly registered accounts with no prior viewing history or user interactions. For each platform, the first 100 videos returned by the default search function were screened for eligibility. Video titles, links, and relevant metadata were recorded at the time of data collection.

All searches were completed on the same day to reduce temporal variations in search results and platform content. All searches were performed using the platforms’ default video ranking without applying any additional filters. Searches were conducted in Shanghai, China, using the same laptop and internet connection throughout the study. No virtual private network (VPN) or proxy service was used during data collection to avoid potential influences on platform-specific search results.

### Eligibility criteria

Videos were included if they primarily focused on dental implant treatment and were available for public viewing at the time of data collection. Both educational and experience-sharing videos were eligible for inclusion, regardless of the uploader type.

Videos were excluded if they met any of the following criteria: (1) unrelated to dental implant treatment (N1); (2) duplicate videos appearing within the search results (N2); (3) purely promotional or advertising content without substantive educational information (N3); or (4) unavailable for viewing or inaccessible at the time of assessment (N4).

All retrieved videos were screened according to these predefined eligibility criteria before inclusion in the final analysis.

### Data extraction

For each included video, the following information was extracted and recorded: platform, ranking position within the search results, video duration, number of likes, favorites, and comments. Additional characteristics included uploader type, video type, presence of individuals in the video, and whether treatment costs or implant brands were mentioned.

Uploader types were categorized as dentist, healthcare institution, science communicator, commercial account, or general user. The presence of individuals was classified as dentist appearing on screen, non-dentist appearing on screen, or no individual appearing on screen.

All data were extracted manually using a standardized data collection form developed for this study.

### Evaluation preparation

Before content evaluation, the URLs of all eligible videos were recorded sequentially immediately after completion of the search process. Each video was subsequently accessed through its recorded URL rather than by repeating platform searches. This approach minimized potential changes in search rankings caused by dynamic recommendation algorithms and improved the reproducibility of the evaluation process. The recorded URLs were retained in the study dataset to facilitate transparency and reproducibility.

### Pilot study

Prior to formal data collection, a pilot assessment of approximately 50 implant-related videos was conducted to clarify the operational definitions of each domain and ensure consistent interpretation of the scoring criteria before application to the full study sample. The pilot assessment was discussed within the research team, and minor refinements were made to the wording and operational definitions of several scoring items before finalizing the ICS.

### Implant Content Score (ICS) development

To evaluate the completeness of implant-related information, an Implant Content Score (ICS) was developed for this study. The scoring system consisted of 10 content domains: indications (ICS1), risk factors and contraindications (ICS2), diagnostic assessment (ICS3), treatment procedures (ICS4), complications (ICS5), scientific accuracy (ICS6), maintenance and follow-up (ICS7), prognosis (ICS8), professional assessment (ICS9), and comprehensive treatment planning (ICS10). The ICS was developed to represent the core information considered essential for patient education in implant therapy, based on the ITI Treatment Guides [11], and was further refined through repeated discussions within the research team.

### Implant Content Score (ICS) Scoring

A domain was considered present only when the corresponding topic was explicitly addressed in the video according to the predefined scoring criteria. Each domain was scored as either present (1 point) or absent (0 points), resulting in a total score ranging from 0 to 10. Higher scores indicated more comprehensive coverage of implant-related information. The complete scoring definitions for all ICS domains, the study dataset containing the original video URLs, and the accompanying variable codebook are provided in Supplementary Dataset S1 to facilitate transparency and reproducibility.

### Global Quality Scale (GQS) scoring

Overall educational quality was assessed using the Global Quality Scale (GQS), a validated five-point instrument widely used to evaluate online health information. GQS scoring was performed according to the original published criteria, with scores ranging from 1 to 5, where higher scores indicated greater educational quality, usefulness for patients, and comprehensiveness of information.

### Content evaluation

All content evaluations were performed independently by a single investigator (K.Y.), an oral and maxillofacial surgeon with clinical experience in implant dentistry, using predefined assessment criteria. The use of a single evaluator was intended to maintain consistent application of the predefined scoring criteria throughout the study. Each ICS domain was scored dichotomously as either present (1) or absent (0), thereby improving consistency in the application of predefined criteria.

### Statistical analysis

Statistical analyses were performed using IBM SPSS Statistics version 29.0 (IBM Corp., Armonk, NY, USA).

Categorical variables were summarized as frequencies and percentages, whereas continuous variables were presented as median and interquartile range (IQR) because the data were not normally distributed.

Differences in the reporting frequencies of individual ICS domains among platforms were evaluated using the chi-square test. Comparisons of video duration, ICS total scores, and GQS scores across platforms were performed using the Kruskal–Wallis test. Associations between video characteristics and information quality were assessed using Spearman rank correlation analysis.

All statistical tests were two-tailed, and a *P* < 0.05 was considered statistically significant.

## Results

### Video selection

A total of 300 videos were retrieved from the three social media platforms, including Bilibili, Douyin, and Xiaohongshu, with the first 100 search results screened from each platform. After applying the predefined inclusion and exclusion criteria, 250 videos were included in the final analysis.

**Fig. 1:**
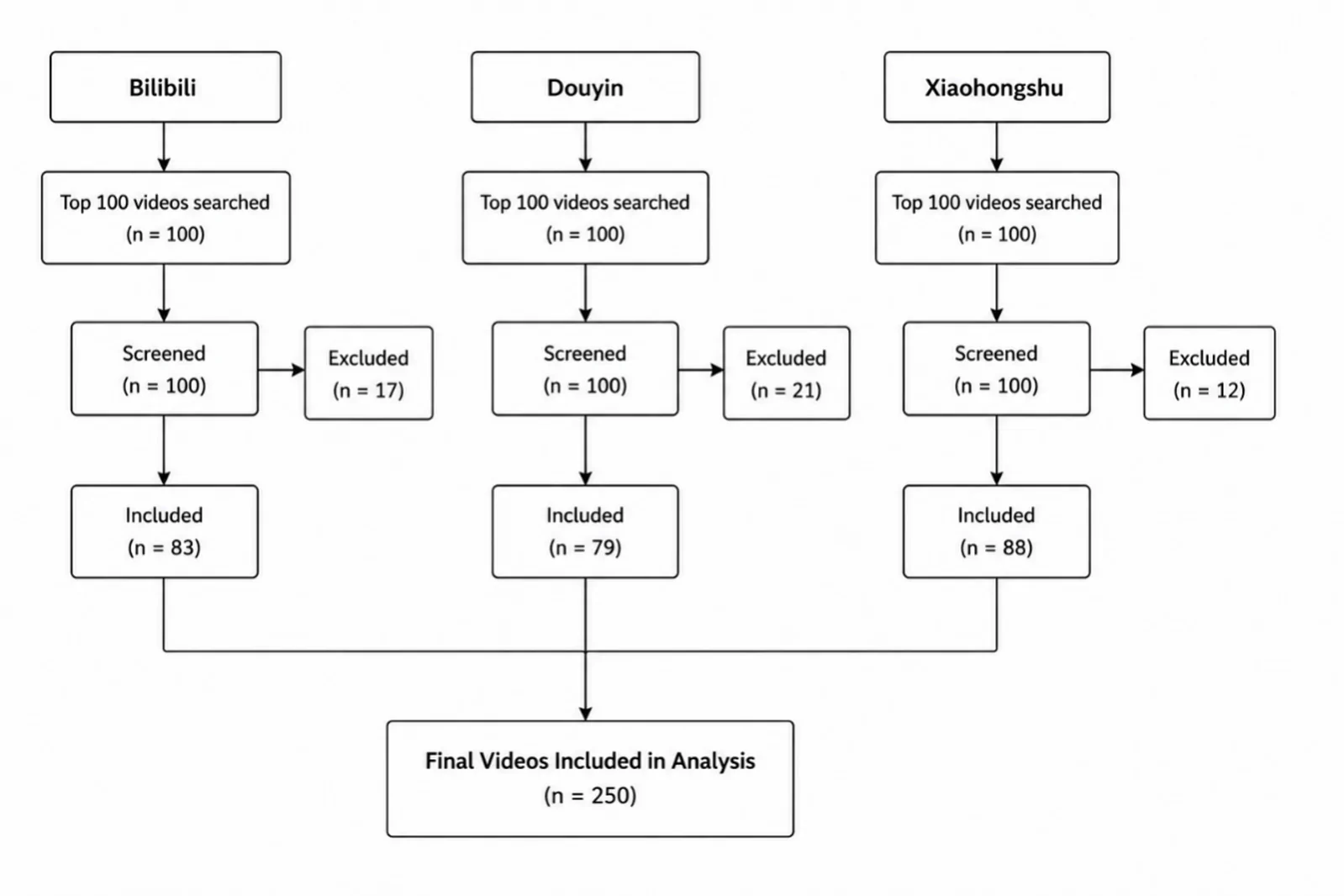
Flow diagram of video selection

Among the 50 excluded videos, 26 were unrelated to dental implant treatment, 22 were duplicate videos, 1 was classified as purely promotional content without educational quality, and 1 video was unavailable for viewing. Ultimately, 83 videos from Bilibili, 79 videos from Douyin, and 88 videos from Xiaohongshu were included in the study. The video selection process is illustrated in Fig. 1.

Flowchart illustrating the identification, screening, eligibility assessment, and inclusion of implant-related videos from Bilibili, Douyin, and Xiaohongshu. A total of 300 videos (100 from each platform) were identified using the search term “种植牙” (“dental implant”). After applying predefined inclusion and exclusion criteria, 250 videos were included in the final analysis.

### Characteristics of included videos

The characteristics of the included videos are presented in Table 1. A total of 250 implant-related videos were analyzed, including 83 videos from Bilibili, 79 from Douyin, and 88 from Xiaohongshu.

**Table 1:** Characteristics of included videos by platform

Variable	Bilibili (*n* = 83)	Douyin (*n* = 79)	Xiaohongshu (*n* = 88)	*P* value
Video duration, sec	217 (122–373)	59 (44–88)	74 (34–136)	< 0.001
Likes	138 (42–491)	234 (38–909)	66 (18–391)	—
Comments	50 (8–258)	28 (3–167)	12 (3–63)	—
Favorites	112 (29–393)	118 (16–579)	28 (10–247)	—

Data are presented as median (IQR)

*P* values were calculated using the Kruskal–Wallis test

Significant differences in video duration were observed among the three platforms (*P* < 0.001). Bilibili videos were substantially longer than those on Douyin and Xiaohongshu, with a median duration of 217 s compared with 59 and 74 s, respectively. In contrast, Douyin videos were generally shorter and more concise.

User engagement metrics showed numerical differences across platforms. Douyin videos tended to receive higher numbers of likes, whereas Bilibili videos attracted more comments and favorites. Xiaohongshu videos generally showed lower engagement metrics than those on the other two platforms.

### Content characteristics of implant-related videos

The content characteristics of the included videos are summarized in Table 2. Among the evaluated content domains, scientific accuracy (ICS6) was the most frequently observed component, being present in 97.2% of videos. Treatment procedures (ICS4) were also commonly discussed, appearing in 55.6% of videos.

**Table 2:** Content characteristics of implant-related videos

ICS Item	Overall *n* (%)
ICS1 Indications	79 (31.6%)
ICS2 Risk factors/Contraindications	59 (23.6%)
ICS3 Diagnostic assessment	67 (26.8%)
ICS4 Treatment procedure	139 (55.6%)
ICS5 Complications	62 (24.8%)
ICS6 Scientific accuracy	243 (97.2%)
ICS7 Maintenance and follow-up	48 (19.2%)
ICS8 Prognosis	47 (18.8%)
ICS9 Professional assessment	31 (12.4%)
ICS10 Comprehensive treatment planning	22 (8.8%)

In contrast, several clinically important topics were infrequently addressed. Information regarding indications (ICS1), diagnostic assessment (ICS3), risk factors and contraindications (ICS2), and complications (ICS5) was present in fewer than one-third of the videos. Furthermore, maintenance and follow-up (ICS7), prognosis (ICS8), and professional assessment (ICS9) were discussed in less than 20% of the videos.

Notably, comprehensive treatment planning (ICS10) was the least frequently reported content domain, appearing in only 8.8% of videos.

### Comparison of information quality across platforms

Significant differences in overall information quality were observed among the three social media platforms (Table 3). The median ICS total score was highest for Bilibili [4 (2–5)], followed by Xiaohongshu [3 (2–4)] and Douyin [2 (1–3)] (Kruskal–Wallis *H* = 30.92, *P* < 0.001). Significant differences were also observed in GQS scores (*H* = 37.54, *P* < 0.001), with Bilibili showing the highest educational quality, followed by Douyin and Xiaohongshu.

**Table 3:** Comparison of Overall Information Quality Scores Across Social Media Platforms

Variable	Bilibili (*n* = 83)	Douyin (*n* = 79)	Xiaohongshu (*n* = 88)	Kruskal–Wallis H	*P* value
ICS total score	4 (2–5)	2 (1–3)	3 (2–4)	30.92	< 0.001
GQS	3 (3–4)	3 (3–3)	2 (1–3)	37.54	< 0.001

Data are presented as median (interquartile range, IQR). Comparisons among platforms were performed using the Kruskal–Wallis test. All tests were two-tailed, and a *P* value < 0.05 was considered statistically significant

**Fig. 2:**
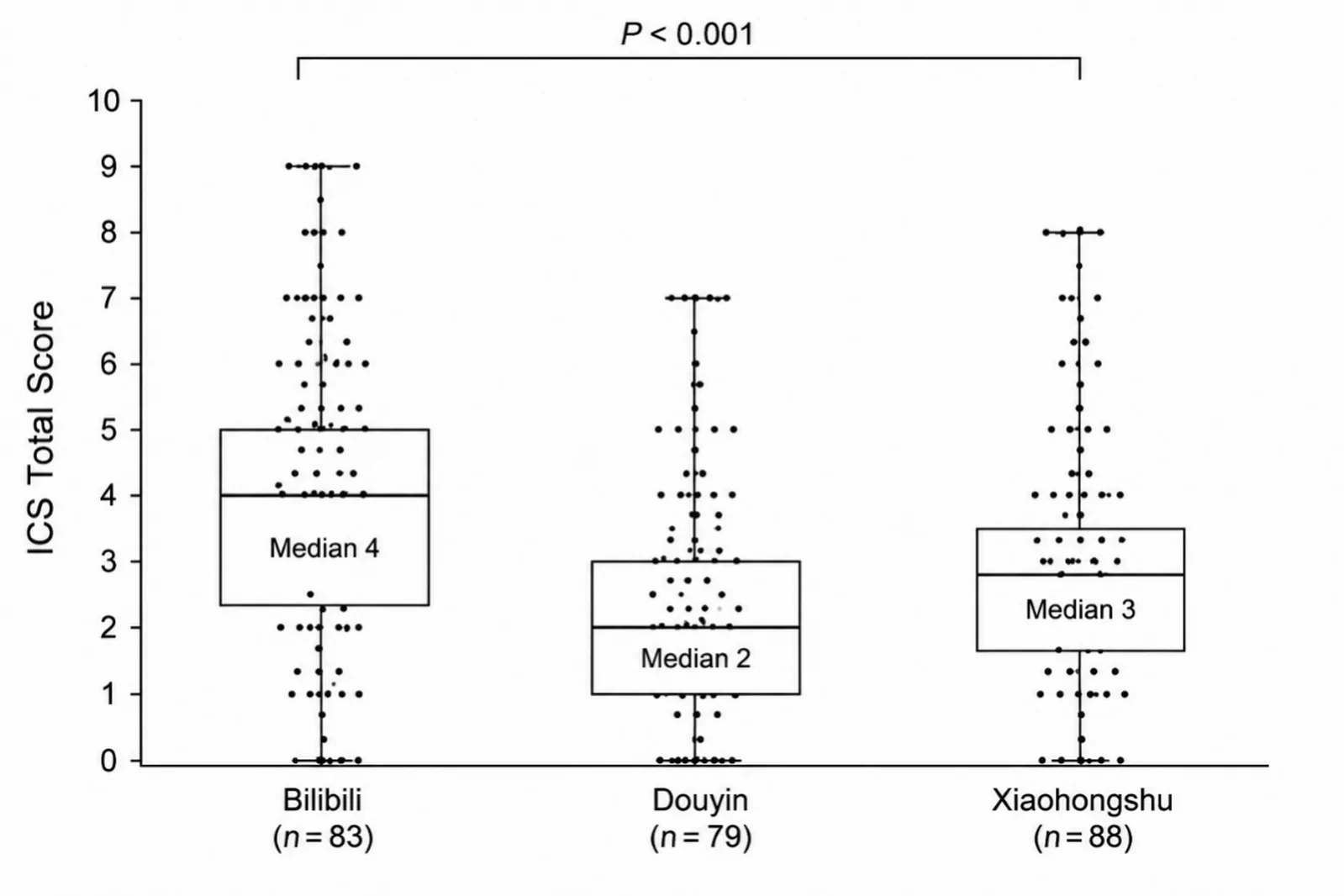
Distribution of implant content score across social media platforms

The distribution of individual ICS components also differed among platforms (Table 4). Significant differences were identified for indications (ICS1, *P* < 0.001), treatment procedures (ICS4, *P* < 0.001), complications (ICS5, *P* < 0.001), prognosis (ICS8, *P* = 0.034), and professional assessment (ICS9, *P* = 0.016). For these domains, Bilibili generally demonstrated higher reporting frequencies than Douyin and Xiaohongshu. The distribution of ICS total scores across platforms is illustrated in Fig. 2.

Boxplots with jittered data points illustrate the distribution of Implant Content Score (ICS) total scores for implant-related videos from Bilibili (*n* = 83), Douyin (*n* = 79), and Xiaohongshu (*n* = 88). The horizontal line within each box represents the median, the box boundaries represent the interquartile range (IQR), and the whiskers extend to 1.5 × IQR. Individual data points represent included videos. Overall differences among platforms were assessed using the Kruskal–Wallis test (*P* < 0.001).

**Table 4:** Comparison of ICS components across platforms

ICS Item	Bilibili (*n* = 83)	Douyin (*n* = 79)	Xiaohongshu (*n* = 88)	*P* value
ICS1 Indications	37 (44.6%)	7 (8.9%)	35 (39.8%)	< 0.001
ICS2 Risk factors/Contraindications	19 (22.9%)	23 (29.1%)	17 (19.3%)	0.325
ICS3 Diagnostic assessment	29 (34.9%)	17 (21.5%)	21 (23.9%)	0.116
ICS4 Treatment procedure	64 (77.1%)	21 (26.6%)	54 (61.4%)	< 0.001
ICS5 Complications	30 (36.1%)	6 (7.6%)	26 (29.5%)	< 0.001
ICS6 Scientific accuracy	81 (97.6%)	78 (98.7%)	84 (95.5%)	0.424
ICS7 Maintenance and follow-up	19 (22.9%)	17 (21.5%)	12 (13.6%)	0.252
ICS8 Prognosis	23 (27.7%)	10 (12.7%)	14 (15.9%)	0.034
ICS9 Professional assessment	15 (18.1%)	3 (3.8%)	13 (14.8%)	0.016
ICS10 Comprehensive treatment planning	9 (10.8%)	6 (7.6%)	7 (8.0%)	0.721

Data are presented as n (%). *P* values were calculated using the χ² test

In contrast, no significant differences were observed for risk factors and contraindications (ICS2), diagnostic assessment (ICS3), scientific accuracy (ICS6), maintenance and follow-up (ICS7), or comprehensive treatment planning (ICS10) (all *P* > 0.05). Notably, comprehensive treatment planning was infrequently addressed across all three platforms, with reporting rates below 11%.

### Factors associated with information quality

The associations between video characteristics and information quality are presented in Table 5. Spearman correlation analysis revealed a moderate positive correlation between video duration and ICS total score (*r* = 0.490, *P* < 0.001), indicating that longer videos tended to provide more comprehensive implant-related information.

**Table 5:** Association Between Video Characteristics and Information Quality

Variable	Spearman *r*	*P* value
Video duration (seconds)	0.490	< 0.001
Likes	0.055	0.389
Favorites	0.096	0.131
Comments	0.053	0.405

Spearman rank correlation analysis between video characteristics and ICS total score

In contrast, no significant correlations were observed between ICS total score and the numbers of likes (*r* = 0.055, *P* = 0.389), favorites (*r* = 0.096, *P* = 0.131), or comments (*r* = 0.053, *P* = 0.405).

## Discussion

The present study evaluated the quality and characteristics of implant-related videos on three major Chinese social media platforms using both a newly developed Implant Content Score (ICS) and the Global Quality Scale (GQS). Overall, substantial variation in information quality was observed across platforms, with differences identified in both content completeness and educational quality. Although scientific accuracy was frequently observed, several clinically important topics, including complications, maintenance, prognosis, and comprehensive treatment planning, were inadequately addressed. These findings are consistent with previous studies evaluating implant-related content on YouTube and TikTok, which also reported limitations in content completeness, reliability, and educational value [8–10]. Collectively, these findings suggest that while social media platforms may serve as accessible sources of implant-related information, the educational quality and comprehensiveness of such content remain inconsistent.

Bilibili demonstrated the highest scores in both ICS and GQS assessments, suggesting that its implant-related videos generally provided more comprehensive and educationally valuable information. One possible explanation is that the platform supports longer-form educational content, allowing creators to discuss diagnosis, treatment procedures, complications, maintenance, and prognosis in greater detail. In addition, platform-specific characteristics, including creator incentives and audience expectations, may also contribute to differences in information quality. This interpretation is consistent with previous studies showing that longer and more structured health-related videos tend to provide higher-quality educational information [7–9, 12].

A notable finding of this study was the discrepancy between ICS and GQS rankings for Xiaohongshu and Douyin. Although Xiaohongshu videos achieved higher ICS scores than Douyin videos, their GQS scores were lower. This suggests that content completeness and overall educational quality represent distinct but complementary dimensions of information quality, consistent with previous studies emphasizing that online health information quality is a multidimensional construct that cannot be fully captured by a single assessment tool [13, 14].

A possible explanation is that Xiaohongshu contains a substantial proportion of patient-generated experience-sharing content, which may mention multiple aspects of implant treatment but lack systematic organization, evidence-based explanations, or comprehensive educational quality. Social media platforms are increasingly used for sharing personal health experiences, and such content may enhance relatability and user engagement while varying substantially in reliability and educational quality [15, 16]. In contrast, Douyin videos may present information in a more accessible and engaging manner despite covering fewer implant-related content domains. Consequently, videos may achieve favorable educational quality ratings despite providing less comprehensive implant-related information.

These findings highlight the importance of evaluating both content completeness and educational quality when assessing health information on social media platforms. The discrepancy observed in the present study supports the complementary use of ICS and GQS, as combined assessment approaches may better capture the multidimensional nature of online health information quality.

Interestingly, user engagement metrics, including likes, comments, and favorites, were not significantly associated with information quality in the present study. This finding suggests that popularity-based indicators may not reliably reflect educational quality, as user engagement can be influenced by factors beyond informational quality, such as emotional appeal, personal relevance, entertainment value, or platform-specific recommendation mechanisms.

The limited reporting frequency of comprehensive treatment planning was one of the most notable findings of the present study, with this domain being addressed in only 8.8% of included videos. While many videos focused on procedural aspects of implant therapy, few discussed implant treatment within the broader context of overall oral health, functional rehabilitation, or alternative treatment options.

This finding may be related to the characteristics of social media content dissemination, where concise and procedure-oriented presentations are often more suitable for online consumption than comprehensive clinical discussions. However, from a clinical perspective, implant therapy should not be viewed as an isolated intervention. Contemporary implant treatment planning requires consideration of multiple factors, including periodontal status, remaining dentition, occlusal conditions, systemic health, patient expectations, and long-term maintenance requirements [17, 18]. Furthermore, successful implant therapy depends not only on surgical placement but also on appropriate case selection, risk assessment, and long-term supportive care [11, 19].

The limited discussion of these considerations on social media may contribute to an oversimplified understanding of implant therapy among viewers. Clinicians should be aware that patients increasingly obtain implant-related information from social media before consultation. Understanding the strengths and limitations of these online resources may facilitate more effective dentist–patient communication, help clinicians address common misconceptions, and support shared decision-making. Furthermore, these findings may encourage implant specialists and professional organizations to actively participate in developing high-quality, evidence-based educational content to improve public understanding of implant therapy.

This study has several strengths. To our knowledge, it is one of the first studies to systematically evaluate implant-related videos across three major Chinese social media platforms. In addition to assessing overall educational quality using the GQS, we developed the Implant Content Score (ICS) to evaluate the completeness of implant-specific information. The combined use of these instruments provided complementary perspectives on online implant-related information quality and enabled a more detailed comparison of content characteristics across platforms.

Several limitations should also be acknowledged. First, social media content is highly dynamic, and the quality and availability of videos may change over time. Second, only the first 100 search results from each platform were evaluated, which may not fully represent all implant-related content available online. Third, the study focused exclusively on Chinese-language videos and therefore may not be generalizable to other linguistic or cultural contexts. Finally, all content evaluations were performed by a single investigator, and therefore inter-rater reliability could not be assessed. Although predefined scoring criteria were applied consistently throughout the study, evaluator-related bias cannot be completely excluded.

Future studies should further validate the ICS in independent cohorts and evaluate its inter-rater reliability using multiple trained clinicians. Longitudinal studies are also needed to investigate changes in implant-related social media content over time. Additional Chinese platforms, such as WeChat Channels, should be explored, and multilingual comparisons with international platforms such as YouTube may provide broader insights into global patterns of online health information dissemination. Finally, future research should examine how exposure to implant-related social media content influences patient understanding, treatment decision-making, and clinical outcomes.

## Conclusions

The quality of implant-related information on Chinese social media platforms varies substantially. Videos on Bilibili demonstrated the highest levels of content completeness and educational quality, whereas clinically important topics such as comprehensive treatment planning were frequently overlooked across all platforms. The findings further suggest that content completeness and educational quality represent distinct dimensions of information quality and should be evaluated using complementary assessment tools. Improving the quality and comprehensiveness of implant-related educational content on social media, together with greater involvement of dental professionals in evidence-based health communication, may enhance patient understanding and support informed treatment decision-making.

## Supplementary Information

Supplementary Material 1.

## Data Availability

All data generated or analysed during this study are included in this published article and its supplementary information files.

## Abbreviations

GQS: Global Quality Scale
ICS: Implant Content Score
IQR: Interquartile Range
